# 3D-printed flexible organic light-emitting diode displays

**DOI:** 10.1126/sciadv.abl8798

**Published:** 2022-01-07

**Authors:** Ruitao Su, Sung Hyun Park, Xia Ouyang, Song Ih Ahn, Michael C. McAlpine

**Affiliations:** 1 Department of Mechanical Engineering, University of Minnesota, Minneapolis, MN 55455, USA.; 2 Computer Science and Artificial Intelligence Laboratory, Massachusetts Institute of Technology, Cambridge, MA 02139, USA.; 3 Sustainable Technology and Wellness R&D Group, Korea Institute of Industrial Technology, Jeju-si, Jeju-do 63243, Republic of Korea.; 4 School of Mechanical Engineering, Pusan National University, Geumjeong-gu, Busan 46241, Republic of Korea.

## Abstract

The ability to fully 3D-print active electronic and optoelectronic devices will enable unique device form factors via strategies untethered from conventional microfabrication facilities. Currently, the performance of 3D-printed optoelectronics can suffer from nonuniformities in the solution-deposited active layers and unstable polymer-metal junctions. Here, we demonstrate a multimodal printing methodology that results in fully 3D-printed flexible organic light-emitting diode displays. The electrodes, interconnects, insulation, and encapsulation are all extrusion-printed, while the active layers are spray-printed. Spray printing leads to improved layer uniformity via suppression of directional mass transport in the printed droplets. By exploiting the viscoelastic oxide surface of the printed cathode droplets, a mechanical reconfiguration process is achieved to increase the contact area of the polymer-metal junctions. The uniform cathode array is intimately interfaced with the top interconnects. This hybrid approach creates a fully 3D-printed flexible 8 × 8 display with all pixels turning on successfully.

## Teaser

OLED arrays were fully 3D-printed under ambient conditions to produce a flexible information display technology.

## INTRODUCTION

Organic light-emitting diode (OLED) displays are competitive alternatives to liquid crystal displays (LCDs) due to their characteristics of self-emission, high contrast ratio, full viewing angle, power efficiency, and mechanical flexibility (*1*). Typically, in commercial OLED displays, the active layers (or emitting layers) are thermally evaporated to achieve high uniformity and resolution (*2*–*4*). Printing methods are being actively investigated because of the potential for scaling up to large panel displays and reduction of material waste (*5*). Fully printed displays in which all functional components are fabricated by printing methods could lead to futuristic concepts, such as higher dimensional form factors, displays interwoven with soft robotics for electroluminescent body parts (*6*) and three-dimensionally (3D) structured pixel matrices for holography (*7*). Yet, methodologies to fully print OLED displays require overcoming several challenges to transfer the materials and processes to printing platforms. Previous publications that reported “fully” printed OLEDs relied on spin coating or thermal evaporation to deposit certain components and create functional devices (*8*–*12*). OLED active layers could be printed in place of evaporated or spin-coated counterparts (*10*, *12*, *13*), but the electrodes and interconnects require sputtering or vapor deposition of materials such as metals, metal oxides, and graphene to achieve high electrical conductivity and optical transmittance (*14*–*16*). In addition, plasma-based deposition processes are typically required to produce oxide encapsulating layers with low moisture diffusion to improve device lifetime (*17*, *18*). Innovations in the material systems, device configurations, printing processes, and design modalities are required to comprehensively print next-generation displays in a manner that is completely untethered from conventional microfabrication manufacturing facilities.

Extrusion-based 3D printing has emerged as a method to assemble a wide palette of materials with varying viscosities, with the possibility of transcending the planar limitations of conventional microfabrication and catalyzing the emergence of truly 3D active electronic devices (*19*, *20*). 3D-printed electronics have features such as spatially structured architectures (*21*), direct side-by-side assembly of hybrid devices (*22*), seamless interweaving of diverse materials (*23*), and printability on moving, free-form, and deformable surfaces (*24*, *25*). Recent progress in 3D printing electronic materials has moved beyond passive conductors toward active electronic materials, including semiconducting quantum dots and conjugated polymers for optoelectronic devices such as LEDs and photodetectors (*22*, *26*). The ability to formulate optoelectronic devices entirely on 3D printing systems enables an unconventional design space for displays and image sensors. However, further development is required in layer-stacking mechanisms and printing methodologies for interconnected optoelectronic arrays with individually addressable pixels.

One obstacle lies in the nonuniformity of extrusion-printed active layers, owing to the directional mass transport within the printed droplets that is driven by the capillary flow during solvent evaporation (*27*, *28*). A second challenge is the creation of repeatable and stable polymer-metal junctions between the active layer and the cathode using the 3D printing approach at room temperature (*29*, *30*). Last, the printed cathode structures should present a uniform array of conductors so that electrical interfaces can be established between the individual pixels and spatially structured interconnects. For instance, Christian *et al.* (*31*) recently reported a single OLED that was “fully” fabricated on a 3D printer, but the dispensed top cathode, a Galinstan droplet, did not allow for conformal printing of electrical interconnects, such that the individual device had to be manually wired to an external power source. In addition, the inkjet-printed active layer was not optimized, resulting in weak light emission that was only observed at the edge of the active area.

Here, we report a multimodal 3D printing methodology and device design scheme that leads to fully 3D-printed, highly flexible OLED displays. To solve the printability issue for the electrodes and encapsulation layer, we extrusion-printed functional inks of a wide range of viscosities, in the form of solutions, liquids, pastes, and resins. Specifically, inks based on metallic nanoparticles were extrusion-printed as ring-shaped bottom interconnects to define the pixel positions and sizes, followed by coating the active areas with a conductive polymer to form a composite anode structure. The top cathode array was extrusion-printed with a eutectic liquid metal stabilized by an oxide shell that formed at room temperature via contact with air. Last, a composite paste material formed the top conductive interconnects, creating an individually addressable OLED matrix via intimate extrusion printing of traces along the cathode array.

In addition to extrusion printing, spray printing and mechanical reconfiguration were used to optimize the fabrication of active layers and polymer-metal junctions, respectively. To improve the uniformity of printed active layers, a spray nozzle was integrated on the 3D printer to atomize the inks into droplets on the scale of tens of micrometers. The reduced droplet size translated into a suppressed directional mass transport within the active layer, leading to a more uniform distribution of the electroluminescent polymer. A controllable thickness of the active layer was realized by tuning the ink concentration and spray time for each pixel. Compared to extrusion-printed devices, OLEDs with spray-printed active layers exhibited improved irradiances and lifetimes, attributable to the reduced barrier to charge transport with an enhanced interlayer contact (*32*), and tunable layer thickness (*33*). To create stable polymer-metal junctions on the 3D printing platform, we leveraged the viscoelasticity of the oxide shell wrapping the liquid metal droplet to implement a mechanical compression process to reconfigure its morphology (*34*), yielding an improved contact area for the polymer-metal junctions. The reconfiguration process produced a spatially uniform liquid metal array for interfacing with the extrusion printed top interconnects and was repeatable over a wide range of compression rates.

In summary, OLED displays were realized by seamlessly integrating materials with disparate rheological and electrical properties entirely on a “one-pot” 3D printing platform that united multiple processing modalities including extrusion, spray, and mechanical reconfiguration. This novel device structure and 3D printing approach enabled a proof-of-concept demonstration of a highly flexible and fully functional 8 × 8 OLED display with all pixels turning on successfully.

## RESULTS

### Device configuration and multimodal 3D printing

**Fig. 1.**
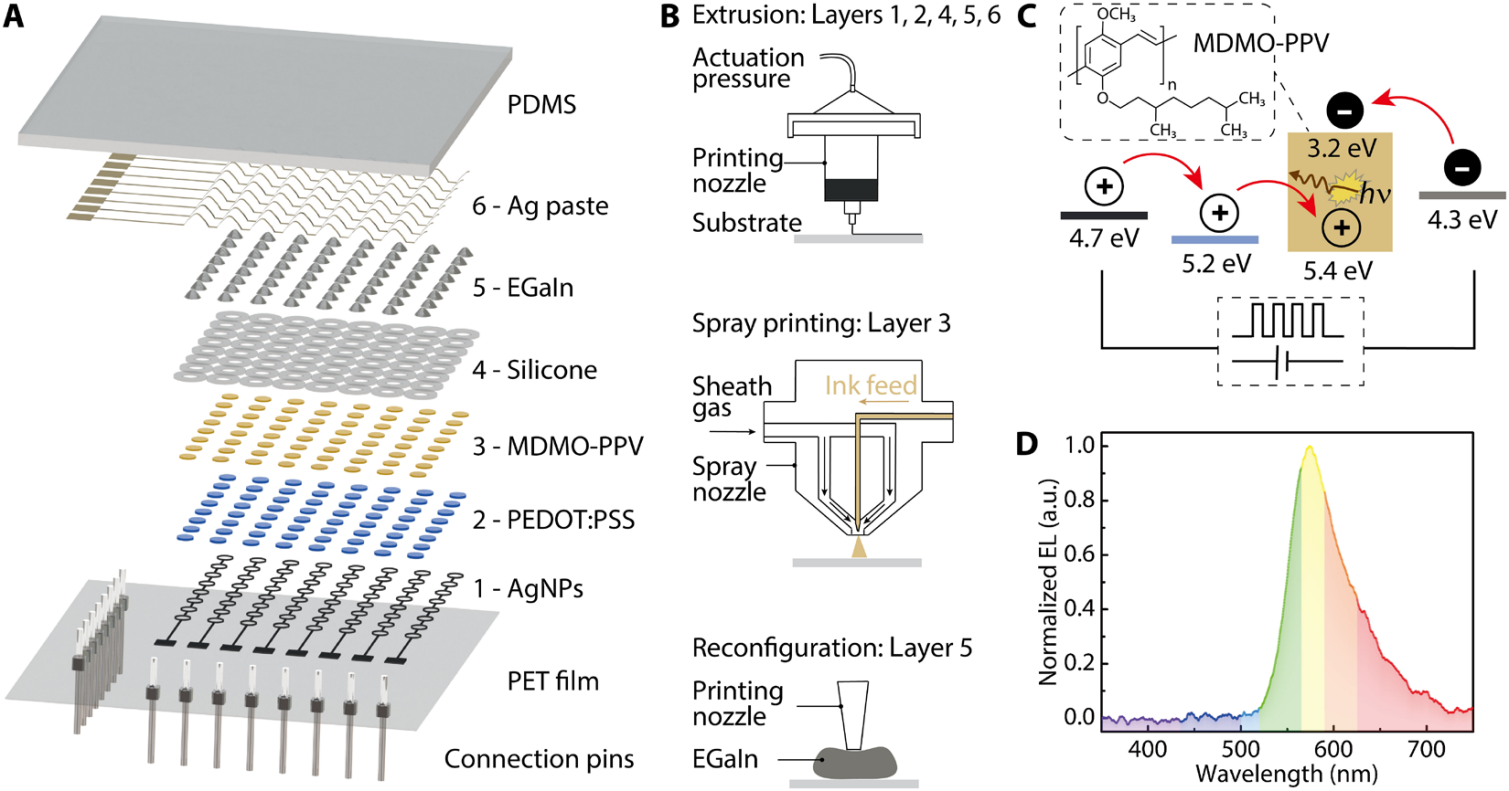
Schematic of the fully 3D-printed OLED display and printing methodology. (**A**) Exploded view of the OLED display demonstrating its layer-by-layer structure. Layers 1 through 6 are 3D-printed components. The OLED display was printed on PET films that were mounted with electrical connection pins and encapsulated with PDMS. (**B**) Schematic demonstrating the methods for printing and reconfiguring each component of the OLED display. (**C**) Energy band diagram of the OLED showing the transport and recombination of the charge carriers under a constant or pulsed external voltage. From left to right, the four materials are AgNPs, PEDOT:PSS, MDMO-PPV, and EGaIn. The inset image displays the molecular structure of MDMO-PPV. *hv*, absorbed photons. (**D**) Electroluminescence (EL) spectrum of the 3D-printed OLED. a.u., arbitrary units.

In our design, the functional components of the OLED display comprised six layers that were 3D-printed on polyethylene terephthalate (PET) flexible films (Fig. 1A). From bottom to top, the constituent layers included the following: (i) bottom interconnects printed with silver nanoparticle (AgNP) inks, which defined the layout of the matrices and created contact pads for connecting to external driving circuits; (ii) a thin-film array of the conductive polymer, poly(3,4-ethylenedioxythiophene) polystyrene sulfonate (PEDOT:PSS), which coated the underlying silver rings to form a composite anode structure, providing enhanced current injection into the active layer while maintaining optical transmittance for light extraction; (iii) a thin-film array of the electroluminescent polymer, poly(2-methoxy-5-(3′,7′-dimethyloctyloxy)-1,4-phenylenevinylene) (MDMO-PPV), which is the active layer within which light emission occurred via the recombination of electrons and holes; (iv) a silicone-based insulation layer that separated bottom silver structures from the top conductive materials, printed to cover all underlying conductive materials and only exposing the active areas of the OLEDs to the cathode; (v) an array of eutectic gallium-indium (EGaIn) droplets, which were subsequently reconfigured to form the top cathode structure; and (vi) an array of top interconnects intimately interfaced with the EGaIn array to complete the terminal contact pads. Last, the device was encapsulated with polydimethylsiloxane (PDMS) that was cast into an extrusion-printed silicone mold, forming a flexible and transparent top layer after cross-linking.

We implemented a multimodal 3D printing method to accommodate the distinct properties of the constituent inks and enable the targeted functions of the OLED displays (Fig. 1B). Because the performance of OLEDs is sensitive to the uniformity and thickness of the active layer, we used a spray printing method to deposit the MDMO-PPV thin films. Compared to extrusion-printed active layers, spray-printed MDMO-PPV films showed improved layer uniformities and controllable thicknesses. Atomization at the nozzle orifice due to friction between the high-speed sheath gas and the low-speed ink broke the fluid into microdroplets, generating an atomized flow stream toward the substrate (*35*). The other five layers were deposited via a pneumatically actuated extrusion printing process (movie S1). For the cathode, the high surface tension of the extruded liquid metal droplets led initially to an incomplete metal coverage with the active layer (*36*). Consequently, the printing nozzle was used to reconfigure the morphology of the EGaIn array to create an enhanced contact area for the polymer-metal junctions.

The electroluminescent polymer MDMO-PPV was selected as the emitter of the 3D-printed OLEDs (Fig. 1C) because of its high performance and stability for both light emitting and photovoltaic applications (*37*–*39*). With the selected material system and successful charge injection enabled by the alignment of energy bands (retrieved from literature) (*40*–*43*), the targeted recombination of charge carriers in the MDMO-PPV layer was achieved. Light emission was attained from the OLED displays under constant or pulsed bias for applications in lighting and information display. The 3D-printed OLEDs demonstrated an emission peak of ca. 574 nm (Fig. 1D), which corresponds to the 2.2-eV bandgap of MDMO-PPV (*41*).

### Spray-printing MDMO-PPV active layer

**Fig. 2.**
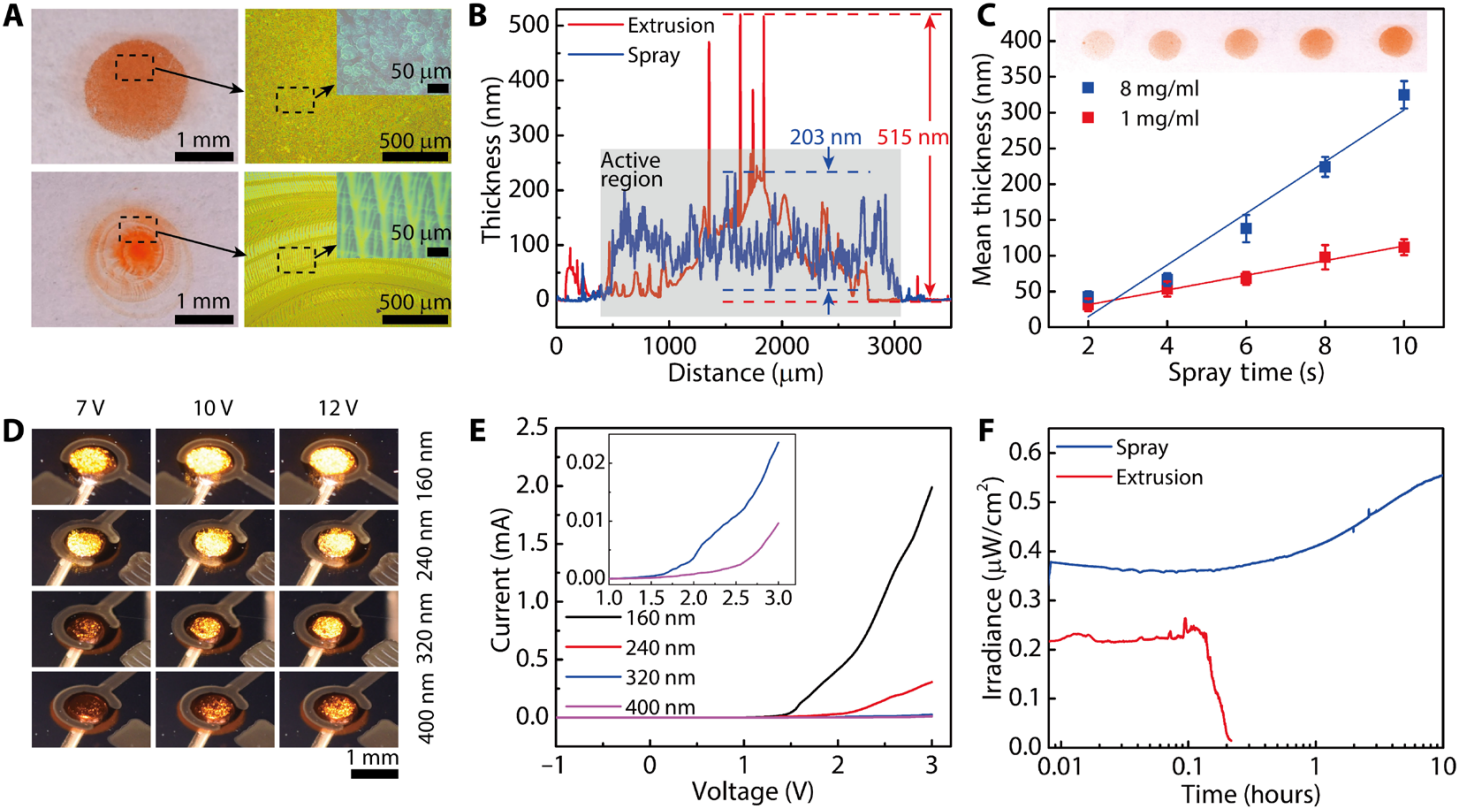
Spray printing of MDMO-PPV as the active layer of the OLEDs for improved layer uniformity and device performance. (**A**) Optical images of MDMO-PPV layers on the macro and micro scales. Circular layers in the top and bottom rows were deposited by spray and extrusion printing, respectively. Photo credit: Sung Hyun Park, University of Minnesota. (**B**) Surface profiles of two MDMO-PPV layers that were spray- and extrusion-printed. Ink concentrations of 1 and 8 mg/ml were used for extrusion and spray printing, respectively. (**C**) Plot of the relationship between the mean thicknesses of MDMO-PPV layers and spray times under two different concentrations. The thicknesses were linearly fitted. *n* = 5. The inset image shows the spray-printed active layer with an ink concentration of 8 mg/ml. Photo credit: Sung Hyun Park, University of Minnesota. (**D**) Optical images of spray-printed MDMO-PPV LEDs under operation. Devices in each row had the same spray-printed thickness, and devices in each column were operated under the same voltage. Photo credit: Sung Hyun Park, University of Minnesota. (**E**) *I*-*V* curves of spray-printed MDMO-PPV LEDs that have active layers with different thicknesses. The inset plot magnifies the *I*-*V* curves of 320 and 400 nm in the range of 1 to 3 V with the same axis titles and units as the main plot. (**F**) Plot of the relationship between the irradiance and operation time for spray- and extrusion-printed MDMO-PPV LEDs that were injected with a current of 30 μA. The two tested devices have a similar mean active layer thickness of ca. 300 nm.

Achieving uniformity in printed active layers is critical to the reliability and tunability of device performance. Uncontrolled extrusion printing of active layers can lead to an uneven accumulation of materials in the drying solution droplet due to the capillary flow during solvent evaporation, especially near the pinned contact line (*27*). Other factors such as irregular droplet contours and adhesion between active polymers and the substrate can lead to undesirable material concentration near the droplet center that are substantially smaller than the active areas of the OLEDs. Overall, the lack of uniformity in the active layers caused large variations in light emission across the active regions within the same batch of devices, signifying the need for alternatives to extrusion printing for the fabrication of large-scale display devices. Therefore, we exploited a spray printing method to deposit MDMO-PPV to improve the uniformity of the active layers. The spray nozzle was integrated into our printing system, whereby the ink was atomized at the orifice when a high relative speed was created between the near-static ink and the pressurized sheath gas. The atomized droplets had diameters in the range of 30 to 50 μm and rapidly evaporated after impacting the substrate, resulting in suppressed mass transport in the lateral direction. In the spray-printed active region, microdroplets were uniformly distributed across the target area and a substantial reduction in the thickness variation was observed (Fig. 2A). In addition, the spray nozzle mounted on the 3D motion stage enabled the formation of dot patterns and continuous lines with feature sizes as small as 1 mm on both planar and 3D surfaces (fig. S1).

Active regions were printed with similar ink volumes to form circular areas with diameters of ca. 2.5 mm in which the spray-printed layer had a surface peak-to-peak variation of 203 nm with a mean thickness of 94 nm and an SD of 37 nm. By contrast, the extrusion-printed layer exhibited a surface peak-to-peak variation of 515 nm with a mean thickness of 87 nm and an SD of 51 nm. The contrast in surface profiles elucidated the notably improved surface morphology of the spray-printed active layer (Fig. 2B). The improved layer quality facilitated more intimate contacts between adjacent layers and promoted the transport and recombination of electrons and holes across different layers. Improved brightness and uniform light emission were observed, attributable to the enhanced uniformity. This was partially verified by the increased emission areas and irradiances of the spray-printed OLEDs compared with the extrusion-printed devices (fig. S2, A and B). The controllability of active layer thicknesses was tested by varying the cumulative spray time and ink concentration. The mean thickness of the MDMO-PPV layer increased linearly as a function of the spray time for the given ink concentrations (Fig. 2C). Changes in ink concentrations induced variations in viscosity and hence in the spray behavior (*44*). Hence, the two ink concentrations exhibited different film deposition rates for the spray-printed active layers.

The layer thickness affects the resistance and electroluminescent efficiency and therefore the light output. By controlling the thicknesses of the active layers, the emission properties of the OLEDs can be tuned. The OLEDs under a given input voltage demonstrated an obvious brightness increase as the layer thicknesses decreased (Fig. 2D). The current-voltage (*I*-*V*) curves of spray-printed OLEDs with an active layer thickness of 160 nm exhibited a reduced turn-on voltage and a comparable electrical resistance to devices with thicker active layers (Fig. 2E). For a thin active layer, the resistance to charge injection was relatively small, such that a lower voltage was required to generate emission. Devices with a spray-printed active layer thickness below 160 nm were susceptible to short-circuiting. As the layer thickness increased, the recombination efficiencies of electrons and holes increased because of the increased diffusion length, but a higher input voltage was required to inject the charge carriers. Therefore, as the sprayed layers became thicker, the light emission became dimmer under the same applied bias, although the brightness could be increased with higher operating voltages (Fig. 2, D and E).

For extrusion-printed devices, there was a higher probability of the presence of defects such as pinholes or incomplete polymer-metal junctions in the active layer due to its nonuniformity, which affected the stable operation of the OLEDs. A 10-hour operation test at 30 μA showed that the light emission from the extrusion-printed device dimmed at an early stage of the test, whereas the spray-printed device exhibited an improved operational stability (Fig. 2F). For spray-printed OLEDs, the irradiance exhibited a rapid initial decay followed by a gradual rise, which agreed with the typical trend of PPV-based OLEDs fabricated using solution-processable methods such as spin coating (*45*). A common explanation relates this behavior to the charge mobility variation as the active material undergoes an improvement in crystallinity and mobility during the initial “annealing” stage, followed by a mobility loss due to the long-term degradation. Accordingly, the charge-injection balance was first deteriorated by the mobility increase and then improved by the loss in mobility (*45*, *46*).

### Liquid metal reconfiguration for cathode arrays

As in our previous studies (*22*, *26*), we selected EGaIn as the cathode material that was printed at room temperature to form a metal-polymer junction with the MDMO-PPV. EGaIn is ideal in ambient conditions because of its chemical stability and low toxicity. With a work function of −4.3 eV and an electrical resistivity of 2.94 × 10^−5^ ohms·cm (*43*), EGaIn has electrical properties comparable to typical cathode metals such as aluminum, allowing for efficient injection of electrons (*47*). Because the deposition of EGaIn is a solvent-free process, redissolution or contamination of the active layer is avoided (*48*). EGaIn provides the targeted electrical performance without requiring thermal or electromagnetic sintering, thereby minimizing potential degradation of the 3D-printed OLEDs. EGaIn was previously used as a back contact that could be conveniently applied on top of fullerene-based active layers of organic photovoltaic cells in lieu of evaporated aluminum (*30*). It has also been recently demonstrated for the electrical characterization of different organic monolayers via a facile formation of polymer-metal junctions (*29*, *49*). When applied to 3D printing systems to create cathode arrays for OLEDs, directly extruded EGaIn droplets form a limited contact area with the underlying polymer layer because of the large surface tension of the metallic ink. The initial near-spherical morphology of the EGaIn droplets also causes displacement and deformation of the cathode structure after contacting extrusion-printed top interconnects, generating irregular OLED active areas and pixel patterns.

**Fig. 3.**
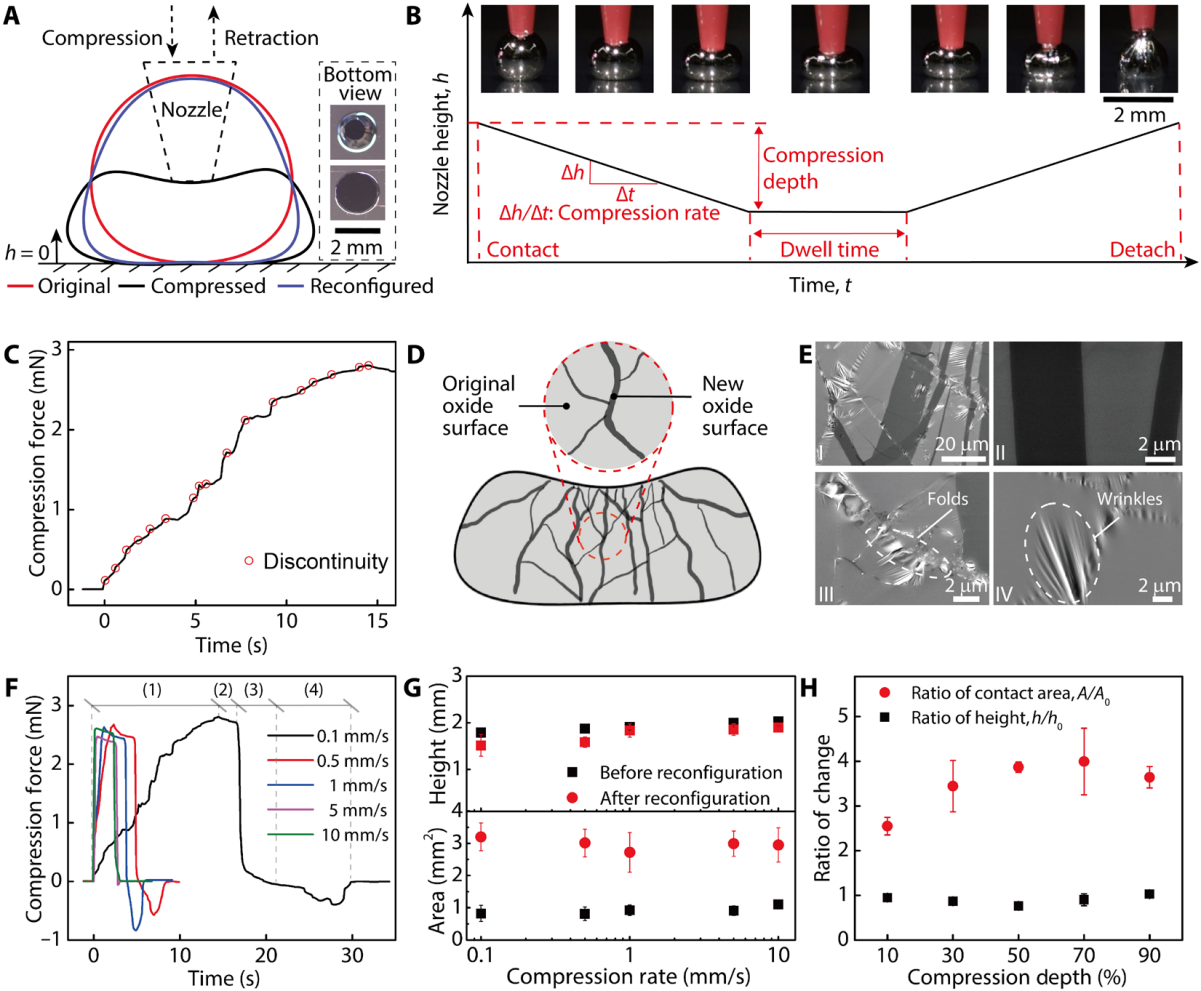
Mechanical reconfiguration of printed EGaIn droplets. (**A**) Schematic of morphology variation of one EGaIn droplet during mechanical reconfiguration with a tapered polypropylene nozzle mounted on the 3D printer. The inset images show the bottom views of one EGaIn droplet before and after reconfiguration. Photo credit: Ruitao Su, University of Minnesota. (**B**) Schematic *h-t* curve depicting the motion of the nozzle during EGaIn reconfiguration. The inset images show the side views of one EGaIn droplet during different stages of the reconfiguration. Photo credit: Ruitao Su, University of Minnesota. (**C**) Compression force versus time curve demonstrating an increasing trend with discontinuities for the force applied to the EGaIn droplet as the nozzle moved downward. (**D**) Illustration of the formation of new oxide surfaces during surface ruptures. (**E**) SEM micrographs demonstrating features on the surface of EGaIn droplets after reconfiguration. (I) Image at lower magnification showing the coexistence of several features. (II) A zoom-in view of the boundaries between the original and new oxide surfaces created by the surface ruptures. (III) A zoom-in view of folds of the oxide surface formed during the retraction of the nozzle. (IV) A zoom-in view of the wrinkles formed on the oxide surface during surface relaxation. Image credit: Ruitao Su, University of Minnesota. (**F**) Complete force-time curves demonstrating the four stages during reconfiguration and the high repeatability of this process for a wide range of compression rates. (**G**) Plots of variations of the morphological metrics, including junction contacting area and height of EGaIn droplets, before and after reconfiguration for compression rates spanning three orders of magnitude. *n* = 5. (**H**) Plot of the relationship between the ratio of morphological metrics after and before reconfiguration and varying compression depths. *n* = 5.

To solve these problems, we hypothesized that the contact area and morphology of 3D-printed EGaIn droplets can be mechanically modulated via a reconfiguration process analogous to conventional metal forging (Fig. 3A). On the basis of our design, the reconfiguration would be enabled by the thin oxide surface that rapidly forms on EGaIn droplets after the material is extruded. The oxide is mainly composed of gallium (III) oxide (Ga_2_O_3_) with a thickness in the range of 1 to 3 nm (*50*). Because the metallic core has a low viscosity, the mechanical behavior of the EGaIn droplets is dominated by the oxide surface. During the reconfiguration, a polypropylene nozzle mounted on the printer was used to compress the EGaIn droplets. The motion of the compression nozzle in the vertical direction was programmed by tuning parameters including the compression rate, dwell time, and compression depth (Fig. 3B). Our tests revealed that the morphology of EGaIn droplets varied during different stages of the reconfiguration. After the nozzle was retracted and detached from the EGaIn surface, the EGaIn droplets presented a new morphology characterized by a similar height but an increased contact area with the layer underneath. Therefore, the high malleability of EGaIn droplets proved to be beneficial to shaping the cathode structures via an extrusion-and-compression process.

Originating from the viscoelasticity of Ga_2_O_3_, the oxide surface yields or flows as the local surface stress increases above the yield strength and eventually ruptures (*50*, *51*). The measurement of the compression force showed repeated discontinuities throughout the reconfiguration process, indicating the rupture of the oxide shell during compression (Fig. 3C). The discontinuities were denoted by abrupt changes in the curve slope, which was also evidenced by a discontinuous deformation of the EGaIn droplet while it was being compressed (movie S2). At the ruptured sites, part of the metallic core was exposed to the air and rapidly reoxidized. The new and original oxide skin connected and reconstructed to form a complete oxide shell (Fig. 3D), which was evidenced by the networks of original and new surface patterns on the EGaIn droplets captured with a scanning electron microscope (SEM). In contrast to the “clean” surfaces of original EGaIn droplets (fig. S3), several features coexisted on the surfaces of reconfigured EGaIn droplets (Fig. 3E). Clear boundaries between the original and newly formed oxide surfaces were observed. In addition, relaxation of the oxide surfaces during the nozzle retraction led to the formation of folds and wrinkles. Regardless of the types of features on the reconfigured surfaces, maps of energy-dispersive x-ray spectrometry demonstrated a uniform distribution of the three major constituent elements (gallium, indium, and oxygen) at the inspected sites, which validated the reoxidation of the ruptured sites (fig. S4 and table S1). The measured elemental composition on the oxide surfaces of EGaIn droplets is consistent with previous surface characterizations conducted under similar conditions (*52*). Therefore, the oxide shell wrapping the EGaIn droplets underwent a rupture-and-reoxidation process during reconfiguration, and the cathode structure was “forged” into a new and stable morphology.

The reconfiguration of EGaIn droplets was repeatable in terms of mechanical behavior, junction contact areas, and geometric profiles of the resulting cathode structure. During the interaction with the designed nozzle motion, the force-time behavior of the EGaIn droplets can be divided into four stages: (i) compression stage in which the compression force increased as the droplets were compressed and the oxide surface ruptured, (ii) dwell stage in which the nozzle halted and the compression force decreased because of the gradual relaxation of the compressed oxide skin, (iii) rapid relaxation stage as the nozzle started retracting, and (iv) pulling stage as the nozzle further retracted and adhesion pulled the droplets until detachment occurred. This characteristic behavior was highly repeatable for the motion rate spanning three orders of magnitude and increased the efficiency for reconfiguring the top electrodes (Fig. 3F and fig. S5, A to E). Within the tested range of the nozzle motion rate, the junction contact areas typically increased by ca. 3× while the heights of the EGaIn droplets remained relatively stable, providing a reliable reference for the printing of top interconnects (Fig. 3G). Further, an increasing compression depth expanded the reconfigured junction contact area, which peaked at 70% compression depth of the initial height and decreased as the compression depth further increased because of the adhesion-induced pulling effect (Fig. 3H). The heights of the reconfigured droplets remained mostly unchanged under different compression depths. The dwell time had no noticeable effect on either the contact area or the height of the reconfigured EGaIn droplets, although a gradual relaxation behavior of the oxide surface was observed during this stage, in accordance with previous studies (fig. S5F) (*50*).

### Flexible OLED displays

**Fig. 4.**
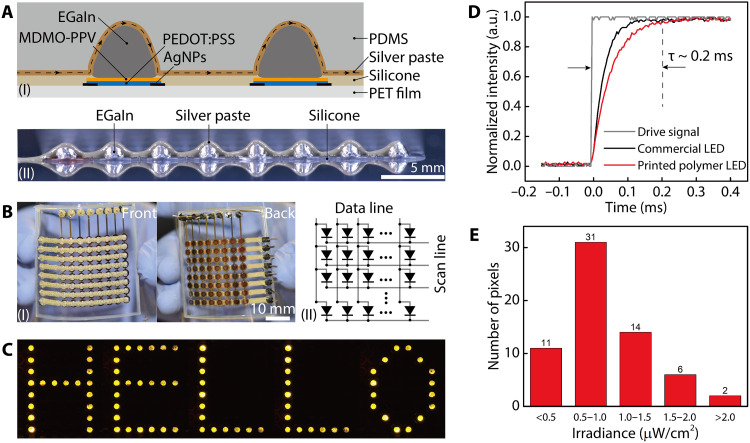
Printing top interconnects and characterization of the 3D-printed OLED display. (**A**) (I) Cross-sectional illustration of two interconnected OLEDs in the display with the top interconnects conformally printed over the reconfigured EGaIn cathodes. (II) Side view of one row in the OLED display before PDMS encapsulation was applied. Photo credit: Ruitao Su, University of Minnesota. (**B**) (I) Images of a completed OLED display, the light emission of which was viewed from the backside. (II) Schematic circuit and driving mechanism of the OLED display. Photo credit: Ruitao Su, University of Minnesota. (**C**) Image of the word “HELLO” while the text scrolled on the 8 × 8 OLED display. Photo credit: Ruitao Su, University of Minnesota. (**D**) Transient characteristics of the 3D-printed OLED and a commercial AlGaInP-based LED. (**E**) Histogram plot showing the irradiance distribution of the 64 pixels in the LED display when each pixel was injected with a current of 10 mA.

To print interconnects on top of the EGaIn array, the reconfiguration nozzle was preloaded with a conductive paste formulated with an epoxy matrix and micro-sized silver fillers. With the unique capability of 3D printing to deposit spatially structured circuits, we programmed the toolpaths of the nozzle according to the morphology of the reconfigured EGaIn droplets so that the top interconnects were conformally interfaced with the cathode array (Fig. 4A and movie S3). In this stacking scheme, the top and bottom interconnects were separated by the silicone insulation layer and electrically coupled through each pixel, creating an individually addressable OLED display. Our printing methodology for both thin-film and metallization layers circumvented the need for photomask sets, cleanrooms, or complicated circuit layouts involved in conventional microelectronics fabrication. To secure the EGaIn arrays and create flexible OLED displays, the fabrication was completed by encapsulating the OLED displays with PDMS that was cast within a 3D-printed silicone mold (Fig. 4B and fig. S6). With anodes and cathodes electrically interconnected along the same columns and rows, respectively, the 8 × 8 OLED displays were addressed in a passive manner whereby the two electrode sets were inputted with the data and scan signals (fig. S7). We demonstrated that all pixels of the fully 3D-printed OLED display worked successfully, and information including text and images was scrolled across the display (Fig. 4C and movie S4).

Because each scan line is turned on only for a fraction of one frame time in the passive driving mode, the pixel response time is important in the functioning of passive-matrix LED displays. The transient response of an OLED originates from the traps of electrons and holes in the organic layers that need to be filled following current injection (*53*). The 3D-printed MDMO-PPV OLED demonstrated a pixel response time of ca. 0.2 ms (Fig. 4D), which is on the same order of magnitude as inorganic aluminum gallium indium phosphide (AlGaInP) LEDs and one order of magnitude faster than typical LCDs (*54*). In the tests of text scrolling with a refresh rate of 125 Hz, no visual blurriness was observed in the displayed moving patterns. When scanned with a current of 10 mA, more than 50% of the pixels fell in the irradiance range of 0.5 to 1.5 μW/cm^2^, with a few pixels in the dimmer or brighter domains (Fig. 4E). The variation in pixel irradiance can be further reduced by minimizing the deviation of active layer thicknesses from the mean thickness.

**Fig. 5.**
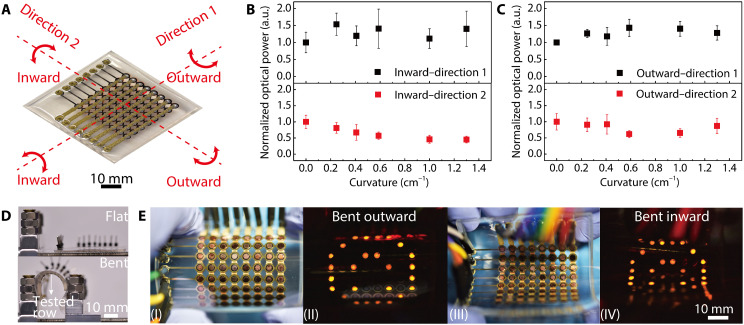
Bending characterization of the 3D-printed flexible OLED display. (**A**) Four different combinations of bending orientations for the OLED display during the bending test. Photo credit: Ruitao Su, University of Minnesota. (**B** and **C**) Optical power of the 3D-printed OLEDs as the bending curvature increased for the four different bending combinations. *n* = 5. (**D**) Images of the OLED display in flat and bent states while mounted on the testing stage. Photo credit: Ruitao Su, University of Minnesota. The tested row of LEDs was under the largest curvature and operated with a current of 10 mA. (**E**) Images of the bent OLED device array while a pattern was displayed on it. (I and II) The backside of the display was bent outward. (III and IV) The backside of the display was bent inward. Photo credit: Ruitao Su, University of Minnesota.

LED displays that can endure large mechanical deformations have important applications in soft electronics, wearable devices, and electroluminescent skins. Our fully 3D-printed OLED displays are highly flexible (movie S5). Yet, the multiple 3D-printed components had disparate mechanical properties, so the stress conditions of different layers vary depending on the applied deformations (Fig. 5A). On the basis of our tests under different bending orientations and curvatures, the OLEDs demonstrated relatively stable performances as supported by the measured optical power from pixels under the largest curvatures (Fig. 5, B to D). For the inward bending along direction 2, which exerted tensile strains to the top interconnects, a declining optical power was observed as the bending curvature increased. This trend was mainly attributed to an increased electrical resistance of the silver-epoxy composite ink when the tensile strain was applied. Silver particles form percolation pathways in the cured top interconnects where the interparticle spacing varies under strains, leading to the observed piezoresistive behavior (*55*, *56*). To test the function of the OLED display during deformation, we designated images to the device and bent it under different conditions. The OLED display was fully functional, and the targeted image was displayed successfully (Fig. 5E). To verify the performance stability of the 3D-printed OLEDs during dynamic deformations, we monitored the output power of single OLEDs undergoing cyclic bending tests (fig. S8). The device exhibited a relatively stable emission over the 2000 bending cycles, suggesting that 3D-printed OLEDs can be potentially used for flexible and wearable displays.

## DISCUSSION

This work presented a multimodal 3D printing methodology that united two different ink delivery methods and a structural reconfiguration process on a common platform to fully 3D-print flexible OLED displays without the need for microfabrication processes. For the active layer, an improved uniformity of the MDMO-PPV films was achieved via a spray printing method that atomized the active material ink into microdroplets, enhancing the controllability of layer thicknesses and device electrical characteristics. The enhanced contact between adjacent layers led to a decreased electrical resistance and an increased illumination uniformity, irradiance, and lifetime. Innovations in material selection and structure design enabled the printing of anodes, cathodes, and interconnects at room temperature. Specifically, a composite anode structure was printed by coating AgNP rings with PEDOT:PSS solutions. Because of the viscoelasticity of the oxide surface wrapping EGaIn droplets, an extrusion-and-compression method was developed to reconfigure the top cathode array, thereby increasing contact areas for the polymer-metal junctions. The reconfigured EGaIn droplets also provided reliable surface profiles on which the top interconnects were conformally printed. Last, the OLED array was encapsulated with PDMS, creating a mechanically robust and flexible display.

From the perspective of material properties, the multimodal printing methodology enabled material depositions and structural modulations with functional inks across a wide range of viscosities and electrical conductivities, overcoming the limitations of printing methods that rely on a single ink delivery modality. The used material system also provided potential solutions to conventional optoelectronic printing methods that face challenges in the printing of electrodes and encapsulation layers at room temperature. The ability to fabricate OLED displays entirely on 3D printing platforms represents a paradigm shift for the printing of optoelectronics, which will affect other types of active devices, such as image sensors, photovoltaics, and computation.

For next steps, the irradiance uniformity of the OLED display will be further improved by optimizing the spray printing conditions and minimizing the thickness variations in the active layers. Methods for improving the printing resolution and scaling down the pixel size will be studied for each layer to create higher-resolution displays. To improve the stability of the device under mechanical deformations, we will develop methods to improve the ink selection and conductivity, particularly for the printing of top interconnects. Last, we will investigate methodologies to integrate the controlling circuits (transistors and capacitors) together with the LED matrices for fully 3D-printed active-matrix OLED displays and other active devices.

## MATERIALS AND METHODS

### Materials

AgNP dispersion [30 to 35 weight % (wt %); part number 736465], PEDOT:PSS solution (0.8 wt %; product number 739316), MDMO-PPV (product number 546461), and EGaIn (product number 495425) were purchased from MilliporeSigma. Room temperature vulcanizing (RTV) silicone (LOCTITE SI 595 CL) for the printing of the insulation layer and encapsulation mold was purchased from Henkel AG & Co. Epoxy-based silver paste (AA-DUCT 916) was purchased from Atom Adhesives LLC. PDMS (SYLGARD 184) was purchased from Dow Inc. PET films (125 μm, MELINEX ST505) were purchased from Tekra LLC.

PEDOT:PSS solution was sonicated for 10 min and passed through a 450-nm polyvinylidene fluoride filter before printing. The active ink was prepared by dissolving MDMO-PPV in toluene and stirring at 1000 rpm for 24 hours. The ink was filtered with a 220-nm polytetrafluoroethylene filter before spray printing. The epoxy-based silver paste was prepared immediately before use by mixing the resin and hardener according to a weight ratio of 1:1.15. PDMS was prepared by mixing the base and curing agent with a weight ratio of 10:1 and defoaming the mixture before casting. The rest of the materials were printed as purchased.

### Spray-printing MDMO-PPV

The spray printing system consisted of one spray valve (781Mini valve with a nozzle size of 0.254 mm, Nordson EFD), one valve controller (ValveMate 7140, Nordson EFD), and one fluid dispenser (Ultimus V, Nordson EFD). MDMO-PPV inks with concentrations of 1 and 8 mg/ml were supplied to the spray valve, and ultra-high purity nitrogen gas (99.998%) was used for both the sheath gas and fluid dispensing gas. The fluid pressure, spray gas pressure, and stroke control knob on the spray valve were set to 31 kPa, 138 kPa, and 1, respectively, to produce a repeatable fine spray. The distance between the spray nozzle and the work surface was maintained at 30 mm, and a PET film–based spray mask was optionally used to pattern the sprayed polymer within the active areas of the OLEDs. The thicknesses of the spray-printed MDMO-PPV layers were analyzed using stylus profilometers (Alpha-Step D-500 and Tencor P-16, KLA).

### EGaIn compression force measurement and imaging

A digital analytical balance (MS304S, Mettler Toledo Inc.) was used to record the compression force during the reconfiguration of the EGaIn droplets. The initial heights of the droplets were measured by recording the position of the nozzle tip when it first contacted the EGaIn surface. The nozzle was mounted on a robotic gantry system (AGS1000, Aerotech Inc). The corresponding compression depth, dwell time, and compression rate were used to program the motion of the nozzle tip. After compression, the reconfigured heights were measured again with the nozzle tip. The contact areas of the EGaIn droplets with the substrates before and after the reconfiguration were analyzed using photographs taken with a camera (Nikon D750).

SEM micrographs of the EGaIn surface were acquired on a JEOL 6500 microscope. The original EGaIn surface was imaged by extruding an EGaIn droplet on an aluminum sample holder that was covered with a piece of carbon tape. The EGaIn surface was imaged after the droplet was reconfigured with a compression depth of 70% of the original height.

### Printed OLED displays

The printing process was conducted on a robotic gantry system (AGS1000, Aerotech Inc) onto which pressure dispensers (Ultimus V, Nordson EFD) and printing nozzles (Nordson EFD) were mounted. Cross-shaped alignment marks were first printed with AgNPs on the PET film to align all layers to the Cartesian coordinate system of the 3D printer. Detailed information about the nozzle size, printing parameters, and curing conditions for each material is listed in table S2. After the top interconnects were printed, the device was stored in a vacuum desiccator for 24 hours so that the ink cured completely. Then, an encapsulation mold was printed with RTV silicone, and electrical connection pins were bonded with the contact pads. Last, PDMS was cast into the silicone mold to encapsulate all printed components. The encapsulated devices were baked in an oven at 75°C for 2 hours before testing.

### *I*-*V* measurements, irradiance, and device operation

The *I*-*V* behavior of printed devices was characterized with a semiconductor device parameter analyzer (Keysight B1500A, Keysight Technologies). The LED light emission was collected using a photodiode power sensor (S130VC, Thorlabs) and a spectrometer (Flame, Ocean Insight). Optical images of the OLEDs were taken with a Nikon D750 camera under an exposure time of 1.3 s and f-number of f/8. The OLED operation was monitored in a dark room with an applied current of 30 μA for 10 hours.

### OLED response time measurement

A pulsed voltage of 12 V was generated via the driving circuit sketched in fig. S7 and applied to one OLED pixel. A commercial cathode-grounded silicon photodiode (SM05PD2A, Thorlabs) was used to detect the light emission from the OLED pixel. A photodiode amplifier (PDA200C, Thorlabs) was used to amplify the generated photocurrent and convert it to a voltage signal. The driving voltage signal and amplified voltage signal were recorded using a digital real-time oscilloscope (TDS 200, Tektronix). The reference AlGaInP LED (TOM-1088CMRL-N3, Oasistek) has a peak wavelength of 640 nm and was powered with a voltage of 2.5 V.

### Passive LED matrix drive for information display

As illustrated in fig. S7, the green subcircuit consisting of an Arduino microcontroller (UNO R3, ELEGOO) and two shift registers (74HC595, Texas Instruments) was used to switch the transistor states. The dark subcircuit that supplies electric power to the OLED display mainly consisted of an external DC power supply (GPC-6030D, GW Instek), eight pairs of field effect transistors (LP0701N3-G, Microchip Technology) and bipolar junction transistors (MJE180G, ON Semiconductor) at the anodes, and eight bipolar junction transistors at the cathodes. During the operation of the OLED display, each scan line was sequentially turned on for 1 ms, and the data signal was synched with the scan signal for each row.
