# Supplementary material for: 3D-printed flexible organic light-emitting diode displays
Source: Sci Adv. 2022 Jan 7;8(1):eabl8798. doi: 10.1126/sciadv.abl8798 (PMC8741182; doi:10.1126/sciadv.abl8798)
Supplement: Supplementary file 1 — Figs. S1 to S8 Tables S1 and S2 [file sciadv.abl8798_sm.pdf]

Science Advances
AAAS

Supplementary Materials for

3D-printed flexible organic light-emitting diode displays

Ruitao Su, Sung Hyun Park, Xia Ouyang, Song Ih Ahn, Michael C. McAlpine*

*Corresponding author. Email: mcalpine@umn.edu

Published 7 January 2022, *Sci. Adv.* **8**, eabl8798 (2022)
DOI: 10.1126/sciadv.abl8798

The PDF file includes:

Figs. S1 to S8
Tables S1 and S2

Other Supplementary Material for this manuscript includes the following:

Movies S1 to S5

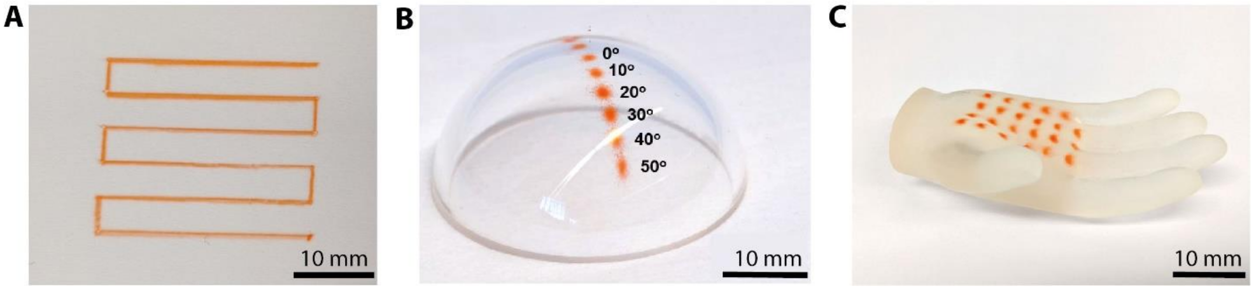

Fig. S1. Various patterns spray printed with MDMO-PPV ink. (**A**) Serpentine continuous lines printed on a planar substrate. Photo credit: Sung Hyun Park, Korea Institute of Industrial Technology. (**B**) Dot array printed on a spherical substrate. Photo credit: Sung Hyun Park, University of Minnesota. (**C**) Dot array printed on the freeform surface of a hand model. Photo credit: Sung Hyun Park, Korea Institute of Industrial Technology.

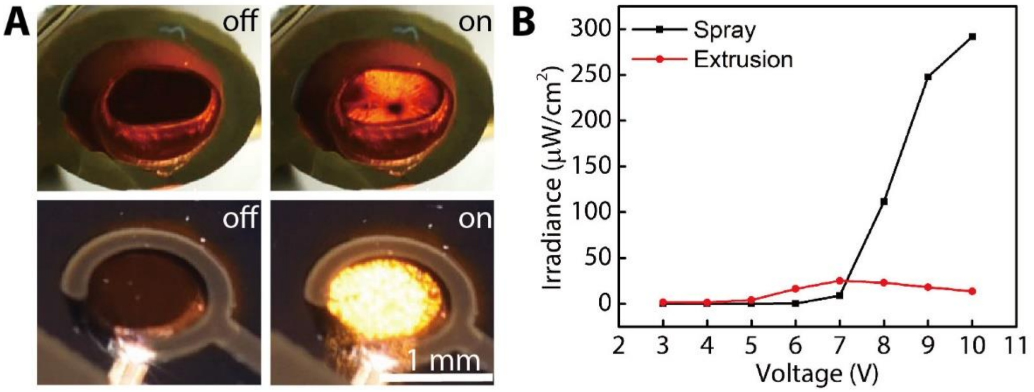

Fig. S2. Comparison of extrusion and spray printed MDMO-PPV LEDs. (**A**) Images of extrusion (top) and spray (bottom) printed LEDs in off and on states. The devices were printed using the MDMO-PPV ink with a concentration of 1 mg/ml. The active layer thickness of the spray printed device is 160 nm, and the same amount of ink was deposited for the active layer of the extrusion printed device. Photo credits: Sung Hyun Park, University of Minnesota. (**B**) Irradiance of extrusion and spray printed OLEDs under varying applied voltage.

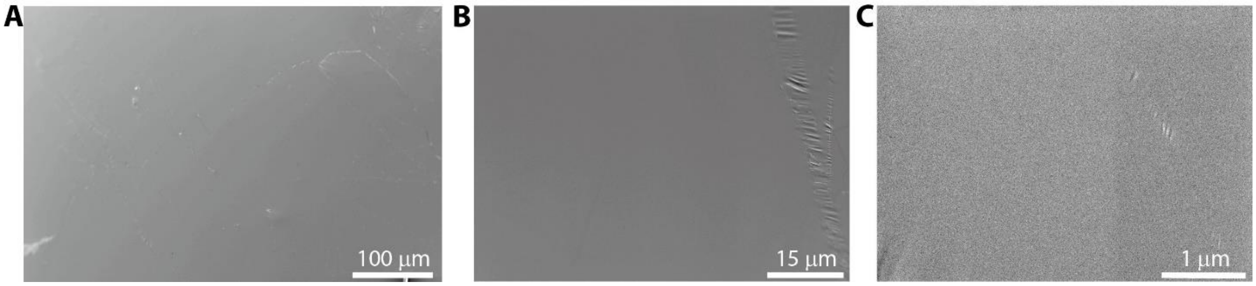

Fig. S3. SEM images at different magnifications of original oxide surfaces on as-extruded EGaIn droplets in which “clean” surfaces can be observed. Photo credits: Ruitao Su, University of Minnesota.

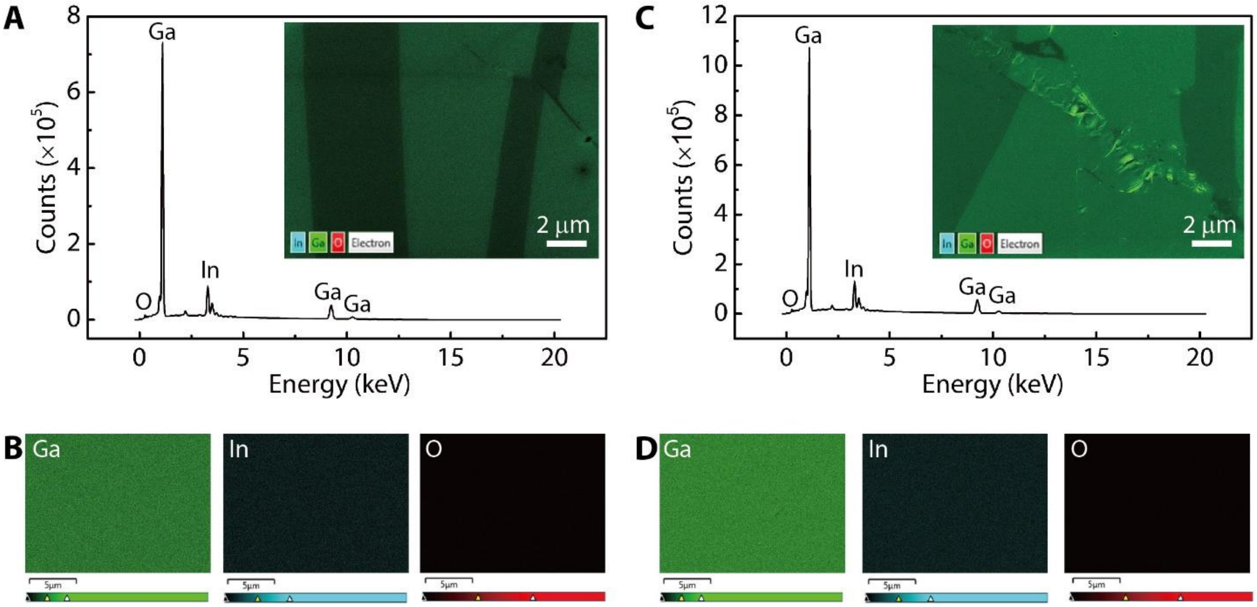

Fig. S4. EDS mapping of two sites on reconfigured EGaIn surfaces. Photo credits: Ruitao Su, University of Minnesota. (**A**) Map sum spectrum and layered SEM/EDS images at Site 1, where boundaries between the original and new oxide surfaces exist. (**B**) Individual elemental distribution maps at Site 1. (**C**) Map sum spectrum and layered SEM/EDS images at Site 2, where folds of the oxide surface exist. (**D**) Individual elemental distribution maps at Site 2.

Table S1. Summary of elemental weight percentages (wt%) at the two inspected sites.

	Site 1			Site 2		
	Ga	In	O	Ga	In	O
wt%	75.4	24.1	0.5	75.2	24.3	0.5

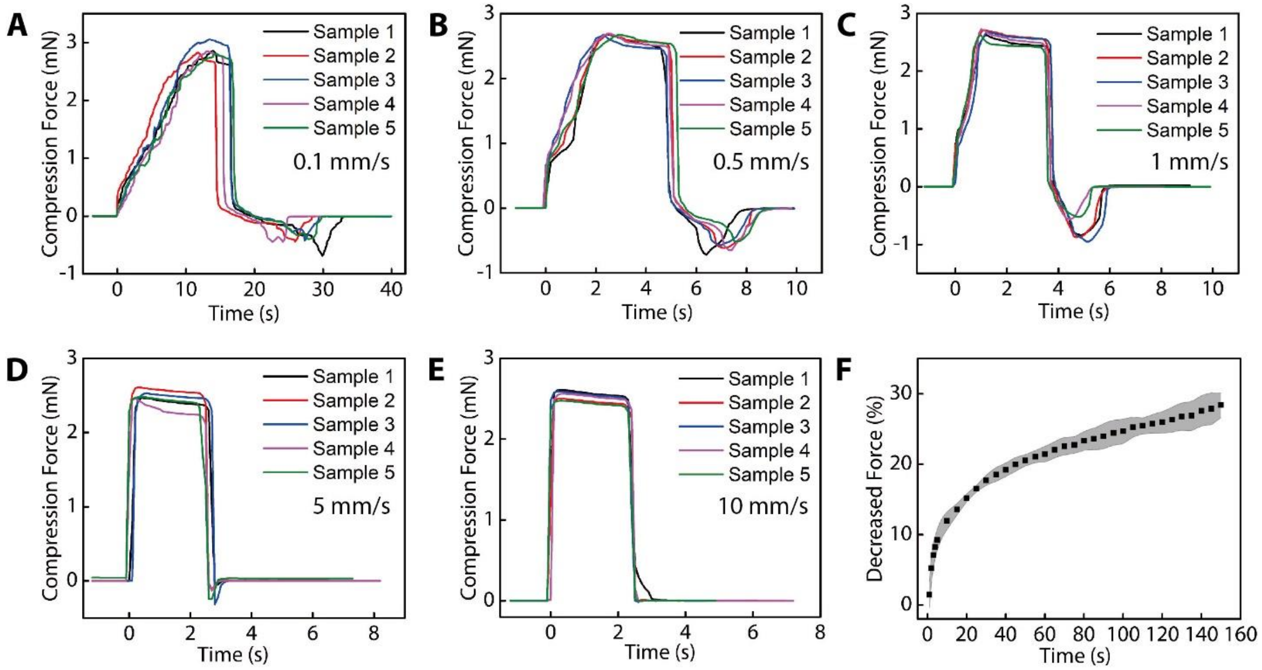

Fig. S5. Characterization of compression force during the reconfiguration of EGaIn droplets. (**A-E**) Force-time curves during EGaIn reconfiguration demonstrated high repeatability for a wide range of nozzle moving rates. The dwell time for all tests was 2 seconds and the compression depth was 70% of the original droplet heights. (**F**) The relaxation of stressed oxide skin yielded a decreasing compression force over time during the dwell stage. $N = 5$.

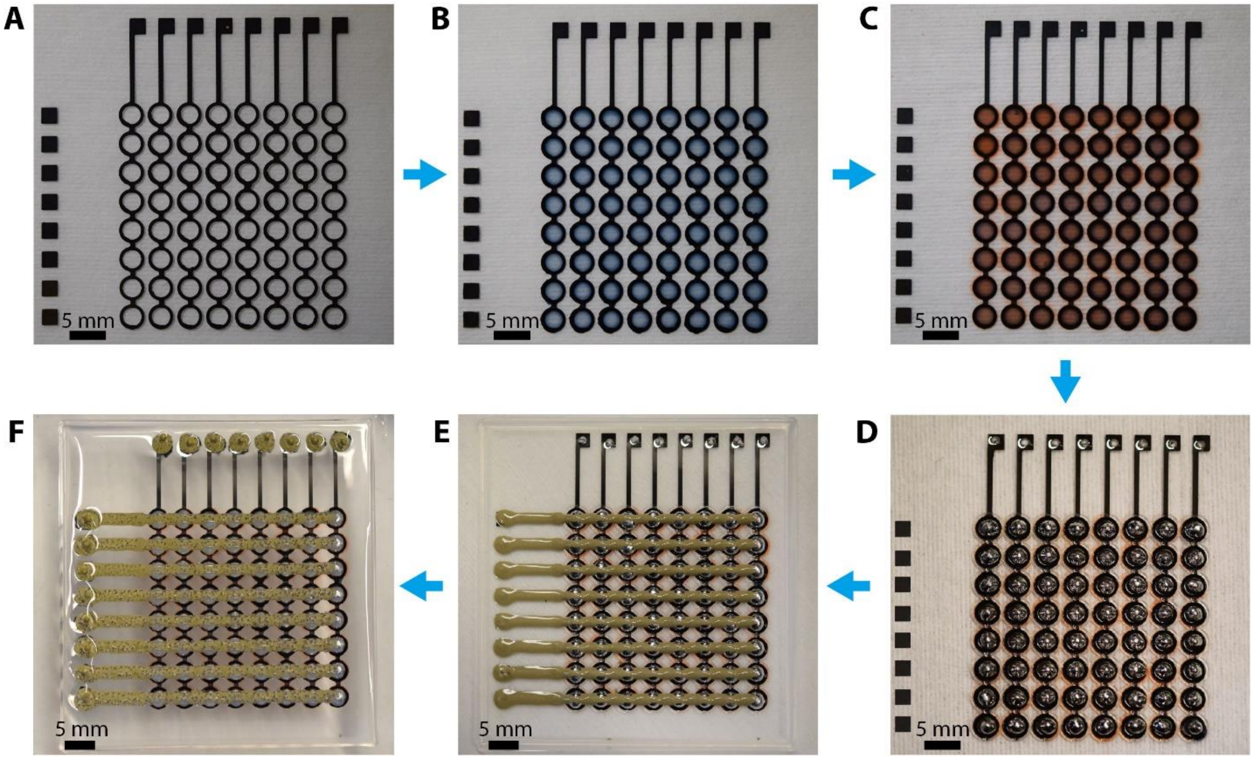

Fig. S6. Major steps in the printing process for OLED displays. Photo credits: Ruitao Su, University of Minnesota. (**A**) Extrusion printed AgNPs form the bottom interconnects for each column. (**B**) Extrusion printed PEDOT:PSS form the bottom anodes. (**C**) Spray printed MDMO-PPV form the emissive layer. (**D**) Extrusion printed silicone and EGaIn form the insulating layer and top cathodes, respectively. EGaIn cathodes were reconfigured with a printing nozzle. (**E**) Extrusion printed silver paste forms the top interconnects for each row. A silicone encapsulation mold was extrusion printed around the OLED display. (**F**) Connection pins were plugged in the silver pads. PDMS was cast in the silicone mold and thermally cured to encapsulate the OLED display.

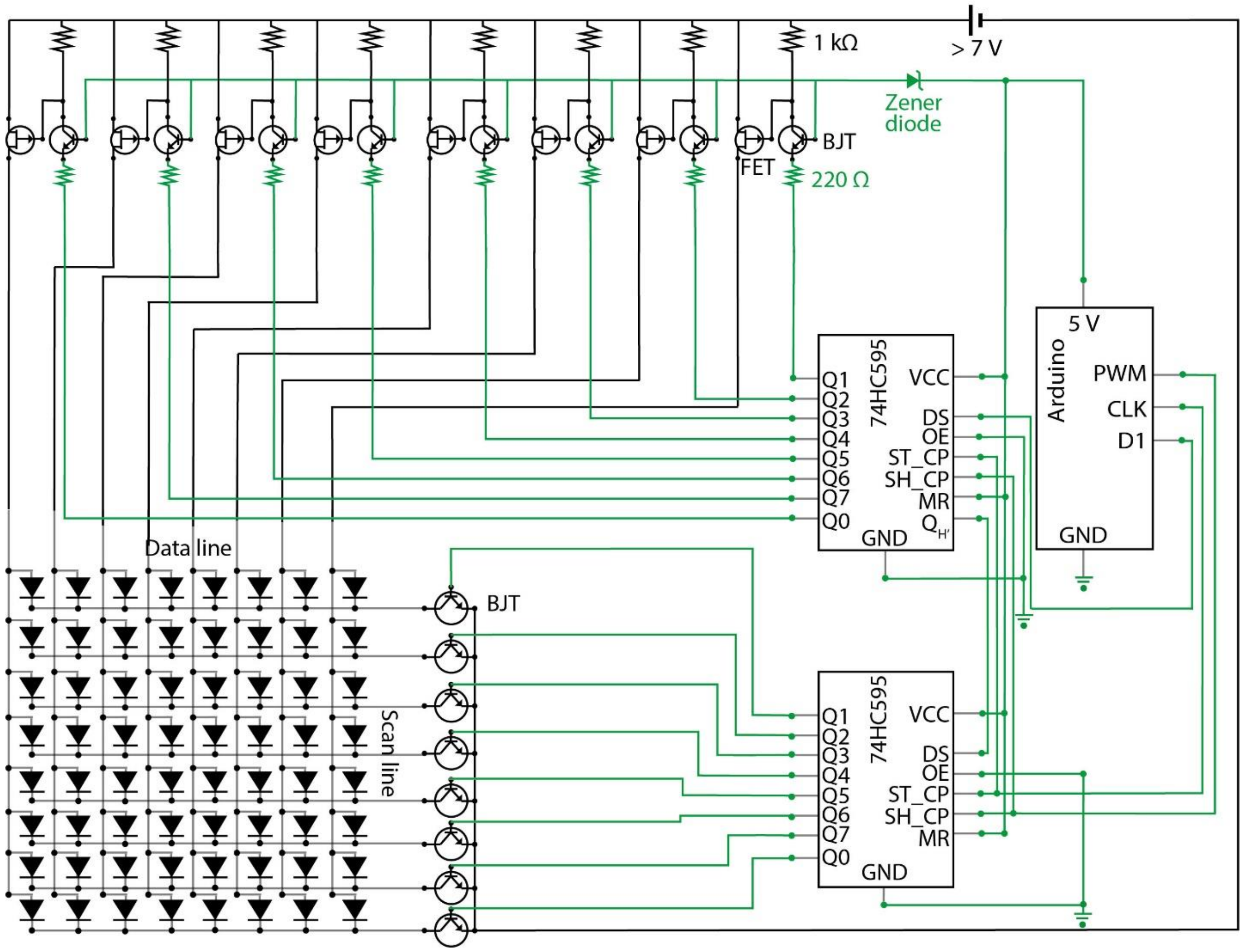

Fig. S7. Diagram of driving circuits for the 3D printed OLED display.

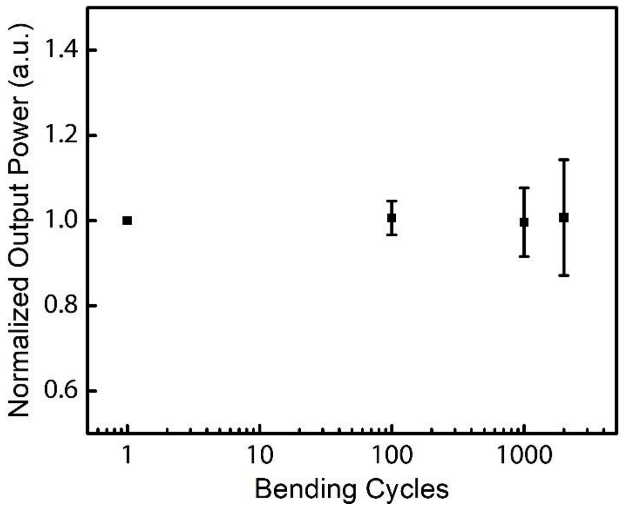

Fig. S8. Device performance stability during cyclic bending tests. The bending curvature was 0.67 cm^{-1} (equivalent to a radius of curvature of 15 mm). Each bending cycle was 3 seconds. The operation voltage was 7 V. $N = 5$.

Table S2. **Printing conditions for the OLED displays.**

Inks	Nozzle Type	Nozzle Inner Diameter (µm)	Pressure (psi)	Speed (mm/s)	Dispensing Time (sec)	Distance to Substrate	Curing Conditions
AgNPs	Stainless steel	100	0.1	5	-	30 µm	120 ℃, 30 min, air
PEDOT:PSS	Stainless steel	100	1.9	5	-	200 µm	120 ℃, 10 min, air
MDMO-PPV	Spray nozzle	254	20 (spray) 4.5 (feed)	-	4-8	30 mm	-
Silicone	Tapered polypropylene	200	30	4	-	200 µm	Room temperature
EGaIn (extrusion)	Tapered polypropylene	200	3.5	-	0.2	2 mm	-
EGaIn (reconfiguration)	Tapered polypropylene	250	-	0.1-10	-	-	-
Silver paste	Tapered polypropylene	250	15	0.5	-	150 µm	Room temperature
PDMS	-	-	-	-	-	-	75 ℃, 2 hours
